# Interest of reference region models to monitor cancer treatment using dynamic contrast enhanced studies

**DOI:** 10.1186/1470-7330-14-S1-P28

**Published:** 2014-10-09

**Authors:** F Frouin, Z Zhang, ME Cohen, M Lefort, A de Cesare, C Pellot-Barakat, O Lucidarme

**Affiliations:** 1 Sorbonne Universités, Paris, France

## Aim

Dynamic Contrast Enhanced (DCE) imaging is an investigative tool to monitor cancer treatments such as antiangiogenic drugs. The difficulty of estimating an appropriate arterial input function in MRI or ultrasound for instance makes the reference region (RR) model very attractive. However it is not applicable to hepatic perfusion studies for which two input functions (hepatic artery and portal vein) have to be considered. A generalization of the RR approach to hepatic DCE studies was thus developed and validated on DCE-CT hepatic studies for which both input functions could be estimated.

## Methods

Our generalization of the RR approach for hepatic studies takes into account two reference regions in addition to the region of interest (or current voxel for parametric imaging). To validate the estimation of the perfusion parameters, numerical simulations were carried out. Ten DCE-CT studies (from five patients with hepatic cancerous lesions observed twice during the course of an antiangiogenic treatment) were also retrospectively analyzed.

## Results

The simulations demonstrated the validity of the new RR model for both noise-free and noisy data. On CT data, perfusion parameters were close when using the two input functions or the new RR model (relative variations being generally less than 10%). This experimental analysis gave also indications for the positioning of the two reference regions.

## Conclusion

This new RR approach is well adapted to estimate perfusion parameters from hepatic DCE studies. It is thus a credible alternative when the input functions are difficult to quantify or out of the field of view.

